# Prospective Study of Nut Consumption and Incidence of Metabolic Syndrome: Tehran Lipid and Glucose Study

**DOI:** 10.3390/nu9101056

**Published:** 2017-09-23

**Authors:** Somayeh Hosseinpour-Niazi, Shabnam Hosseini, Parvin Mirmiran, Fereidoun Azizi

**Affiliations:** 1 Nutrition and Endocrine Research Center, Research Institute for Endocrine Sciences, Shahid Beheshti University of Medical Sciences, Tehran 1985717413, Iran; s.hossainpour@endocrine.ac.ir (S.H.-N.); h.shabnam@yahoo.com (S.H.); 2 Department of Clinical Nutrition and Dietetics, Faculty of Nutrition Sciences and Food Technology, National Nutrition and Food Technology Research Institute, Shahid Beheshti University of Medical Sciences, Tehran 1981619573, Iran; 3 Endocrine Research Center, Research Institute for Endocrine Sciences, Shahid Beheshti University of Medical Sciences, Tehran 1985717413, Iran; azizi@endocrine.ac.ir

**Keywords:** metabolic syndrome, nuts, walnuts, Tehran lipid and glucose study

## Abstract

This study aimed to assess the association of various types of nut per se, and total nut consumption with the incidence of metabolic syndrome (MetS). A 6.2 ± 0.7-year population-based prospective study was conducted among 1265 adults, aged 19–74 years, participants of the Tehran Lipid and Glucose Study. A 168-item semi-quantitative food frequency questionnaire was used to collect information on nut consumption. MetS was defined according to the Joint Interim Statement guidelines and 276 new cases of MetS were identified. Median ± interquartile range of nut consumption was 2.08 (0.88–5.68) servings/week. After adjusting for family history of diabetes, age, gender, smoking, physical activity, fasting serum glucose at baseline, serum high density lipoprotein cholesterol (HDL-C) at baseline, energy intake, fiber, macronutrients, cholesterol intake, fruit, vegetables, dairy products and body mass index (BMI), a statistically significant decrease was observed in MetS in the third (≥5 servings/week) tertile of nuts (odds ratio: 0.68, 95% CI: 0.44–0.91, *p* trend: 0.03) compared with the lowest (≤1 serving/week). Walnut consumption showed a significant, inverse association with MetS risk; associations for other nut varieties were not significant. For each additional serving/week of walnuts consumed, incidence of MetS decreased by 3% (ORs: 0.97 CI: 0.93–0.99), after adjusting for confounding factors. Total nut consumption, especially walnuts, reduces the risk of MetS.

## 1. Introduction

Metabolic syndrome (MetS) characterized by hypertension, central obesity, dyslipidemia, and glucose metabolism disturbances, describes a combination of interrelated genetic, metabolic, and environmental factors [1] and can cause a 2-fold increase in the risk of developing cardiovascular disease (CVD) [2], a 2.5-fold increase in risk of renal disease [3], and a 5-fold increase in risk of type 2 diabetes mellitus (T2DM) [4]. Of note, 20–30% of the adult population worldwide can be defined as having MetS [5]; in Iran, the prevalence is 33.8% [6]. It is predicated that the prevalence of MetS will rise mainly due to a parallel increase in obesity [5], making it essential to address this situation, through dietary intake, which is a modifiable risk factor among other contributing factors (genetic, physical activity, smoking, and education level) associated with MetS [1,7,8]. Among dietary determinants, nuts contain an abundance of healthy fats, fiber, antioxidants, phytochemicals, phytosterols and minerals [9] that can beneficially affect insulin resistance, blood pressure, and dyslipidemia, contributing to their reduction theses inflammatory markers, which are all well-known risk factors for MetS [10,11].

Evidence has emerged from controlled clinical trials showing the beneficial effects of the consumption of nuts (either alone or as part of a Mediterranean diet) on metabolic features among people with MetS [11,12]. To date, there are limited epidemiological studies investigating the association between nut consumption and MetS, and they document conflicting results [10,13,14,15,16,17,18]. Although cross-sectional [13,14,15] studies have shown that nut consumption is inversely associated with obesity and MetS, limited prospective studies investigated the association between nut consumption and MetS; most of them investigate the association between the prudent dietary pattern or a Mediterranean diet enriched with nuts and MetS with conflicting results [10,16]. However, considering other healthy components, besides nuts in prudent dietary patterns, the synergic effects of nuts in the risk of MetS should be taken into account. Only one prospective study reports a reduced risk of MetS with consumption of over two servings/week of nuts over six years of follow-up [10].

Each type of nut has its own particular characteristics [9,19]. To the best of our knowledge, regarding the unique nutrient profiles of nuts and their effects on metabolic status, no previous studies have elucidated the direct association of different types of nuts such as almonds, walnuts, hazelnuts, peanuts and pistachio with MetS risk. Moreover, most of the aforementioned studies were conducted among Mediterranean populations in developed countries [10,11,12], and the long-term potential effects of consumption of different types of nuts on MetS risk among non-Mediterranean populations are not well documented. Thus, this prospective population-based study aimed to investigate the association between the consumption of nuts (total) and its various types including walnuts, almonds, hazelnuts, peanuts and pistachios (each per se) and risk of MetS in a 6.2 ± 0.7-year follow-up study conducted among adults in Tehran, Iran.

## 2. Materials and Methods

### 2.1. Study Population

The present prospective population-based study was conducted within the framework of the Tehran Lipid and Glucose study (TLGS). Details of the TLGS study have been described elsewhere [20]. Briefly, the TLGS is an ongoing prospective study, which aims to prevent noncommunicable diseases through promoting a healthy lifestyle. The TLGS was initiated in March 1999 and follow-up data are being collected every 3 years. In phase I, a multistage, stratified cluster random sampling technique was used to select >15,000 individuals, aged ≥3 years from Tehran’s urban district 13, a group representative of the urban population of Tehran. Phases II, III, IV and V are prospective follow-up studies conducted between 2002–2004, 2006–2008, 2009–2011 and 2012–2015, respectively [20]. The present study was conducted within the framework of the TLGS, during a 6.2 ± 0.7-year follow-up period; baseline examination data of the present study were obtained from phase III TLGS (2006–2008) and the outcome examination data were from phase V (2012–2015).

During the third examination survey of the TLGS (2006–2008), complete data of 12,523 participants (a medical history and physical examinations) were obtained; after this a representative sample of 4920 participants was randomly selected, based on their age and gender, to complete the dietary assessment, a sample size chosen owing to the cost, complexity and time involved in collection of dietary data in a large population. The characteristics of participants who completed the food frequency questionnaire (FFQ) were similar to those of the total population in phase III of the TLGS [21]. Of the 4920 participants in the present study, 3462 agreed to complete the FFQ, of whom, 2895 adults, aged 19–74 years, with complete data (demographic, anthropometric, biochemical, and dietary) were included in the baseline examination of the present study. We excluded participants who had MetS at baseline (phase III) because we aimed to evaluate the incidence of MetS (*n* = 425); also excluded were those who had a recent change in their dietary intake due to serious conditions such as cancer, myocardial infarction, stroke, or other cardiovascular diseases (*n* = 39), participants who under- or over-reported their dietary intakes (<800 kcal/day or >4200 kcal/day, respectively) (*n* = 131), women who were pregnant or lactating (*n* = 106) and participants who had missed follow-up for biochemical, anthropometric and dietary assessments (*n* = 932). Finally, data from 1265 participants were analyzed. Participants with missing data and those who were lost to follow-up were considered non-responders, based on which the response rate of the current study was 44% during the 6.2 ± 0.7-year follow-up. In phase III, there was no difference in the baseline characteristics of responders and non-responders, except that more responders were smokers.

The study protocol was approved by the ethics committee of the Research Institute for Endocrine Sciences (RIES), Shahid Beheshti University of Medical Sciences, and written informed consent was obtained from all participants.

### 2.2. Definition of Metabolic Syndrome

MetS was defined according to the Joint Interim Statement [22], as the presence of three or more of its components: High serum triglyceride (TG) concentrations (≥150 mg/dL or use of anti-hypertriglyceridemia medications); Low serum high-density lipoprotein cholesterol (HDL-C) (<50 mg/dL in women and <40 mg/dL in men); High blood pressure (≥130/85 mmHg or use of anti-hypertensive medications); Hyperglycemia (fasting plasma glucose concentration ≥100 mg/dL or use of anti-hyperglycemic medications); and enlarged abdominal circumference (≥95 cm according to the population- and country-specific cut-off points for Iranian adults of both genders [23]).

### 2.3. Dietary Assessment

Dietary intake information over the previous year was gathered using a validated, semi-quantitative 168-item FFQ [24]. Frequency of consumption of each food on a daily, weekly or monthly basis were documented according to a standard unit or portion size, specified for each food by trained dieticians, during face-to-face interviews; portion sizes of consumed foods converted to grams [25]. Food composition values for macro- and micro-nutrients were obtained from US Department of Agriculture (USDA) food composition tables (FCT) because Iranian FCTs are incomplete. Nutrients intakes were calculated by multiplying the grams consumed of each food in nutrient contents. Total intakes of different types of nuts (almonds, peanuts, pistachios, hazelnuts and walnuts) were each determined as serving sizes. Nut consumption was calculated by summing up weekly consumption of these nuts. In the current study, consumption of various types (per se) and of total nuts in phase III (2006–2008) was used to investigate their associations with risk of MetS. The reliability and validity of the FFQ used for the nut consumption was acceptable (Adjusted correlation coefficient between FFQ and multiple 24 recalls was 0.54 and 0.39; and between the two FFQs was 0.34 and 0.52 in males and females, respectively) [24]. Moreover, there was a reasonable reliability and validity of the dietary patterns, derived from the FFQ, among the population over time.

### 2.4. Biochemical Assessment

At baseline and follow-up, blood samples were drawn after 12–14 h of overnight fasting into vacutainer from all study participants. All blood analyses were done at the TLGS research laboratory on the day of sample collection. Using the enzymatic colorimetric method with the glucose oxidase technique, fasting serum glucose was measured. Enzymatic calorimetric test with glycerol phosphate oxidase was used to measure TG concentrations. HDL-C was analysed after precipitation of apolipoprotein B-containing lipoproteins with phosphotungstic acid. All analyses were performed using commercial kits (Pars Azmoon Inc., Tehran, Iran). Inter- and intra-assay coefficients of variation were both 2.2% for serum glucose, and 2% and 0.5% for HDL-C, and 1.6% and 0.6% for TG, respectively.

### 2.5. Assessment of Other Variables

Weight was measured using a digital scale with participants wearing light clothing and without shoes. Weight was recorded to the nearest 0.1 kg. Height was measured using a stadiometer with participants standing with shoulders in normal alignment and without shoes. Height was recorded to the nearest 0.5 cm. Body mass index (BMI) was calculated as the ratio of weight (kg) divided by height (m^2^). Waist circumference (WC) was measured at the midpoint between the iliac crest and lowest rib and was recorded to the nearest 0.5 cm. Physical activity was assessed using a questionnaire that included a list of common activities of daily life; the frequency and amount of time spent per week on physical activity over the last year were recorded [26]. Physical activity levels were expressed as metabolic equivalent hours per week (METs h/week) [27]. Smoking status was categorized as current smoker or non-smoker. Additional information on age, gender, family history of diabetes, medical history, and current use of medications was obtained and documented using a questionnaire, as reported previously [20]. In the current study, all these variables at phase III (2006–2008) were used as covariates.

### 2.6. Statistical Analysis

Statistical analyses were performed using the Statistical Package for Social Science version 15.0 (SPSS Inc., Chicago, IL, USA). Energy-adjusted consumption of nuts (total) and the various types (each per se) were calculated by the residual method [28]. One-way analysis of variance and Chi-squared test were used to compare characteristics of participants across tertiles of nut consumption. Energy-adjusted means for dietary intake across tertiles of nut consumption were determined using a general linear model analysis of covariance. Multivariable logistic regression was used to estimate the odds ratio (OR) and 95% confidence interval (CI) for MetS across tertiles for nuts and for the various types. Four models were constructed: Model 1 was the crude; Model 2 was adjusted for family history of diabetes, age, gender, smoking status, physical activity level, fasting serum glucose at baseline, and serum HDL-C at baseline; Model 3 was additionally adjusted for dietary variables, including total energy intake, total fiber, percent of protein, percent of carbohydrate, percent of total fat, cholesterol intake, fruit, vegetables, and dairy products. Because BMI might represent a mediator between the association of nuts and MetS, we adjusted for BMI in separate model (model 4); the first tertile was used as a reference. To assess overall trends of the ORs of MetS across tertiles of dietary variables, the median intake of each tertile was used as a continuous variable in logistic regression models. *p* values < 0.05 were considered statistically significant.

We additionally performed stratified analysis by categories of family history of diabetes (yes, no), BMI (<25 and ≥25 kg/m^2^), and age (19–45 and ≥45 years) to estimate ORs of MetS in tertiles of total nut consumption and those of various types of nuts (per se).

## 3. Results

Over the median 6.2 ± 0.7 years of follow-up, 276 new cases of MetS developed among the 1265 study participants. At baseline, the means ± SD of age and BMI were 37.2 ± 11.9 years and 26.4 ± 4.6 kg/m^2^, respectively; 9.5% were smokers, 45.8% were overweight and obese, and 39.2% had academic degrees. Median ± interquartile range (IQR) for consumption of nuts among the study population was 2.08 (0.88–5.68) servings/week. Median ± IQR of nuts were 1.0 (0.4–2.0) for the first, 4.2 (2.6–6.3) for the second and 24.7 (12.1–28.1) grams/week for the third categories of nut consumption, respectively.

Table 1 illustrates the characteristics of participants by tertiles of nut consumption. Participants with higher intakes of nuts were older and were more likely to be smokers. At baseline examination in phase III, fasting serum glucose and serum HDL-C were significantly associated with nut consumption; however, after 6.2 years of follow-up, systolic blood pressure, fasting serum glucose, serum TG, and WC were significantly and inversely associated with nut consumption. No statistically significant associations were observed for gender, physical activity, family history of diabetes, BMI, education levels, occupational status, and use of anti-hyperglycemia, anti-hypertensive and hypolipidemic drugs across tertiles of nut consumption.

No difference in baseline characteristics including age, gender, physical activity, family history of diabetes, BMI, education levels, occupational status, and use of anti-hyperglycemic and anti-hypertensive and hypolipidemic drugs was shown between total nut consumption and its various per se, except for smoking status (Supplementary Materials Tables S1–S5).

Table 2 illustrates the dietary intakes of participants by tertiles of nut consumption. Participants who consumed more nuts had significantly higher intakes of total energy, carbohydrates, protein, fat, polyunsaturated fat (PUFA), total fiber, and fruit. Similarly, with an increasing intake of nuts, intakes of all types of nuts including almonds, peanuts, pistachios, hazelnuts and walnuts increased. Consumption of saturated- and mono-saturated fatty acids, vegetables, cholesterol, meat, poultry, fish, whole grain, legumes, and dairy products did not differ by tertiles of nuts.

The multivariate adjusted ORs (95% CIs) for MetS across tertiles of energy-adjusted consumption of nut and its various types are shown in Table 3. There was a statistically significant decrease in MetS risk among the third (≥5 servings/week) versus the lowest (≤1 serving/week) tertiles of nut consumption in the crude model (OR: 0.55, 95% CI: 0.39–0.77, *p* for trend: 0.002). In the adjusted model for family history of diabetes, age, gender, smoking, physical activity, fasting serum glucose and HDL cholesterol at baseline, participants in the third tertile had a 42% lower risk of MetS (OR: 0.58; 95% CI: 0.42–0.81, *p* for trend: 0.01) compared with those in the reference tertile (first). Further adjustment for dietary intakes attenuated this association (OR: 0.62; 95% CI: 0.45–0.86, *p* for trend: 0.01). This association remained significant after additional adjustment for BMI (OR: 0.68; 95% CI: 0.44–0.91).

Investigation of the associations between consumption of varieties of nuts and the risk of MetS revealed that walnut consumption had a significant inverse association with MetS risk (OR: 0.61, 95% CI: 0.44–0.86, *p* for trend: 0.02). After adjustment for family history of diabetes, age, gender, smoking, physical activity, fasting serum glucose and HDL cholesterol at baseline, a substantial reduction in the risk of MetS was observed (OR: 0.64, 95% CI: 0.47–0.89, *p* for trend: 0.02). Further adjustment for dietary intakes weakened this association (OR: 0.70 95% CI: 0.49–0.94). This association remained significant after additional adjustment for BMI (OR: 0.75; 95% CI: 0.53–0.98). Almonds, hazelnuts, peanuts and pistachios were not associated with risk of MetS. Moreover, for each additional serving/week of walnuts consumed, the incidence of MetS was reduced by 3%, in fully adjusted model, (OR: 0.97, 95% CI: 0.93–0.99).

The ORs (95% CI) of MetS across tertiles of nut consumption (total) and its various types (each per se) stratified by age, family history of diabetes and BMI are shown in Table 4; according to family history of diabetes (yes or no), consumption of total nuts and walnuts was inversely associated with risk of MetS in both groups, associations that were more pronounced in participants with family history of diabetes. Stratified analyses according to age (19–45 or ≥45 years) showed that total nut consumption reduced risk of MetS in both groups, being more pronounced in participants aged ≥45 years; whereas walnut consumption reduced the risk of MetS only in participants aged ≥45 years. Stratified analyses by categories of BMI showed that consumption of total nuts, walnuts and almonds was significantly and inversely associated with reduced risk of MetS only in participants with BMI ≥ 25 kg/m^2^.

## 4. Discussion

In this population-based prospective study with over 6.2 years of follow-up, the consumption of nuts lowered the risk of MetS, independently of confounding factors. Compared with participants who consumed less than one serving/week of nuts, those who had over five servings/week had a 32% lower risk of MetS, an association more pronounced in overweight and obese participants. Walnut consumption was also associated with a 25% reduction in the risk of MetS, an association more pronounced in overweight and obese participants, and participants aged ≥45 years. No association was observed between consumption of almonds, peanuts, pistachios or hazelnuts and the risk of MetS, possibly due to the low consumption of these types of nuts; only almond was inversely associated with MetS among overweight and obese participants.

Results of this study are in agreement with those of other prospective studies investigating the relationship between consumption of nuts and MetS. In a five-year follow-up of the National Health and Nutrition Examination Survey (NHANES), consumption of more than ¼ ounce/day of tree nuts (including walnuts, pistachios, almonds and hazelnuts) reduced the risk of MetS components [15]. A follow-up of the Seguimiento Universidad de Navarra (SUN) study in Spain showed a 34% reduced risk of MetS in women who consumed >2 servings/week of nuts [10]. Moreover, in the Coronary Artery Risk Development in Young Adults (CARDIA) study with 20 years of follow-up, a prudent dietary pattern (including nuts) was associated with a reduced risk of MetS and hypertension, hyperglycemia and dyslipidemia, compared to a Western dietary pattern [29]; these findings are supported by those of a randomized clinical trial in which consuming one serving/day of nuts (15 g walnuts, 7.5 g almonds, or 7.5 g hazelnuts) improved insulin sensitivity among participants with MetS [11]. In addition, previous case-control and cross-sectional studies have shown that frequent consumption of nuts and peanuts is associated with a lower prevalence of risk factors for CVD, T2DM, and MetS [15], as well as obesity [13,14,30].

Most studies showing an inverse association between nut consumption and risk of MetS were conducted among Mediterranean populations in developed countries [10,15,29]. However, existence of other healthy foods, apart from nuts in the Mediterranean dietary pattern, the synergic effects of nuts and these foods in reducing the risk of MetS must also be taken into account. Therefore, considering the healthy contents of the Mediterranean dietary pattern, the beneficial effects reported in the above studies were attained by consuming two servings/week of nuts [10]; amounts less than the five servings/week in the present study. The Atherosclerosis Risk in Communities Study in the United States, however, showed no association between a prudent dietary pattern including nuts and the incidence of MetS [17], a result which may be due to the inclusion of coffee, sweetened beverages, and refined grains in a prudent dietary pattern, which could reduce the net effect of nuts on MetS. In the present study conducted in a Middle Eastern country, the positive effects of consuming over five servings/week of nuts on MetS was confirmed, which may be due to the fact that Iranian dietary patterns are becoming more similar to Western dietary patterns in recent decades [31].

Nut consumption has been recommended by the American Heart Association (AHA) since 2000 [32]. In agreement with this recommendation, dietary advice to promoting a healthy diet by inclusion of nuts (≥5 servings/week), with special focus on walnuts can increase public health. As a nutrient-dense food, frequent inclusion of nuts in the diet provides high amounts of mono- and polyunsaturated fatty acids, vegetable protein, fiber, antioxidants, vitamins, minerals such as magnesium and potassium, and multiple bioactive compounds like phytosterols and polyphenols. All these components of nuts are known to have beneficial effects on metabolic and cardiovascular outcomes [33,34], effects which can be explained by the amelioration of oxidative stress, inflammation and endothelial function, which decreases the risk of hypertension, dyslipidemia, abdominal obesity, insulin resistance and diabetes. In addition, consumption of one serving/day of nuts has been associated with enhanced satiety [35] and better weight management [36].

To the best of our knowledge, previous studies have not examined the direct effect of various types of nuts on MetS risk. In the current study, walnut consumption (median intake 5.8 servings/week) showed a 25% reduction in the risk of MetS, independent of confounding factors. In some interventional studies, walnut consumption has inconsistently been found to be associated with lower risk of MetS, diabetes, and CVD [37,38,39]; however, the association between walnuts and MetS or its components has rarely been investigated in epidemiological studies [40,41]. Data from a cross-over clinical trial among Korean adults with MetS demonstrated that 45 g/day of walnut consumption for 16 weeks improved MetS components, especially fasting blood glucose [37]. In another trial, consumption of 15 g/day of walnuts improved lipid profile of women with MetS [42]. By contrast, a walnut intervention diet used in a South African population showed no significant effect on lipid profiles, inflammatory factors, blood pressure, or serum uric acid concentrations compared with the control diet [38]. Another trial concluded that including walnuts in the diet for a participant group who received intensive lifestyle counseling (LC) provided no additional benefits to lipid profiles, compared with a less intensive LC group [39]. The fairly small sample size, short duration of the intervention (clinically significant outcomes for metabolic factors cannot be determined over a short time), and the low total- and low-density lipoprotein cholesterol concentrations at baseline might partly explain the null findings in the two latter trials [38,39]. Each type of nut has its own particular characteristics. Unlike most nuts that are high in monounsaturated fatty acids (almonds, hazelnuts, pistachios and peanuts), walnuts are composed largely of polyunsaturated fatty acids such as ellagic acid and alpha-linolenic acid, all of which have antioxidant and anti-inflammatory properties that protect against cardiometabolic risk factors and MetS.

In our study, no associations were observed for consumption of the other types of nuts studied (almonds, peanuts, pistachios and hazelnuts) and risk of MetS. However, previous interventional and epidemiological studies have reported beneficial effects of the consumption of almonds, pistachios, hazelnuts and peanuts on cardiometabolic profiles, adiposity, glycemic control, and lipid profiles of individuals with MetS [11,43], pre-diabetes [44], and T2DM [40,45]. The discrepancies between our results and those of the abovementioned studies might be explained by the low consumption of the abovementioned types of nuts among Tehranian adults. In addition, compared with previous study, the sample size of this study is relatively small. For instance, the SUN study, which is a prospective cohort study with Spanish university graduates, identified 567 new cases of MetS among 9887 participants after six years of follow-up [10]. Therefore, further prospective investigations with large sample size are needed to confirm the association between consumption of other varieties of nuts and risk of MetS, as well as the underlying mechanisms.

Some strengths need to be mentioned in this study. First, this study has a population-based prospective design and was conducted in a Middle Eastern country; second, the over 6.2-year follow-up in the study rules out potential seasonal changes in participants’ diets. Third, using a valid and reliable FFQ [24] administered by a trained nutritionist minimized any potential measurement errors. Finally, we adjusted for potential confounding factors, especially total energy intake (usually a confounder of associations between diet and disease in epidemiologic studies) and also took nutrient density into account [28]. Nevertheless, the present study also has a few limitations. First, MetS is heterogeneous; therefore, in addition to dietary factors, factors such as heredity may need to be addressed. Furthermore, despite carefully adjusting for a range of known confounding factors, unknown confounding factors or potential dietary confounders may affect the relationship between nut consumption and MetS. In addition, our findings cannot be inferred to older or younger populations as this study only included adults. Finally, regarding the different types of nuts (other than walnuts) analyzed in this study, the failure to detect any associations between consumption of these types of nuts and MetS may be due to the narrow range of dietary intakes among our participants.

## 5. Conclusions

In conclusion, this population-based prospective study among Tehranian adults with more than six years of follow-up showed that the consumption of nuts, especially walnuts, reduces the risk of MetS. The inclusion of nuts in the diet may be beneficial in reducing the risk of cardiometabolic risk factors and MetS. Further studies are needed to establish the effect of different varieties of nuts on MetS and to clarify the mechanisms involved.

## Figures and Tables

**Table 1:** Characteristics of participants by tertiles of energy-adjusted nuts consumption at baseline (2006–2008) and after 6.2 years of follow-up (2012–2015): Tehran Lipid and Glucose study ^a^.

	Tertiles of Nuts Consumption			
	1	2	3	*p* ^b^
Participants (*n*)	419	425	421	
Age (years)	36.7 ± 11.9	36.2 ± 11.1	38.9 ± 12.6 ^d^	<0.001
females (%)	54.9	54.6	58.7	0.44
Physical activity (MET h-week)	41.1 ± 78.8	35.4 ± 53.1	34.9 ± 51.3	0.27
Family history of diabetes (%)	19.1	19.1	19.7	0.79
Academic degrees (%)	34.7	33.6	36.5	0.80
Occupational status, employed (%)	37.6	41.8	39.5	0.73
Current smoking (%)	13.0	12.2	7.0	0.01
BMI (kg/m^2^)	23.4 ± 4.8	24.4 ± 4.7	22.4 ± 4.2	0.99
Obese (%)	59.8	58.0	59.8	0.84
Anti-hypertensive drugs (%)	75.7	67.1	78.2	0.38
Anti-hyperglycemia drugs (%)	85.7	83.3	83.3	0.98
Hypolipidemic drugs (%)	72.5	72.7	76.5	0.88
Systolic blood pressure (mm Hg)				
At baseline	107 ± 0.6	108 ± 0.6	108 ± 0.6	0.70
After 6.2 years	113 ± 0.7	112 ± 0.7	110 ± 0.7 ^c^	0.02
Diastolic Blood pressure (mm Hg)				
At baseline	72.1 ± 0.4	71.3 ± 0.4	72.0 ± 0.4	0.49
After 6.2 years	76.7 ± 0.5	76.0 ± 0.5	75.8 ± 0.5	0.30
Fasting serum glucose (mg/dL)				
At baseline	91.5 ± 0.6	86.2 ± 0.6	80.2 ± 0.6 ^c,d^	0.04
After 6.2 years	95.3 ± 0.9	93.5 ± 0.8	84.1 ± 0.9 ^c,d^	0.02
Serum triglyceride (mg/dL)				
At baseline	126 ± 3.4	127 ± 3.3	123 ± 3.4	0.68
After 6.2 years	137 ± 4.0	141 ± 3.9	126 ± 3.5 ^c,d^	0.01
Serum HDL-C (mg/dL)				
At baseline	43.0 ± 0.5	43.5 ± 0.5	45.2 ± 0.5 ^c,d^	0.007
After 6.2 years	50.0 ± 0.6	49.6 ± 0.6	51.0 ± 0.6	0.26
Waist circumference (cm)				
At baseline	86.9 ± 0.6	87.3 ± 0.6	86.6 ± 0.6	0.74
After 6.2 years	93.4 ± 0.5	91.9 ± 0.5	90.5 ± 0.5 ^c^	0.04

BMI, Body Mass Index; obese, BMI ≥ 25 kg/m^2^; MET, metabolic equivalent; HDL-C, high density lipoprotein cholesterol; ^a^ Mean ± SE for all these values, except for variables was determined. ^b^ *p* values determined using ANOVA for continuous variables and chi-square test for categorical variables. Significantly different from tertile 1 (Tukey pairwise comparisons in general linear model) *p* < 0.05. Significantly different from tertile 2 (Tukey pairwise comparisons in general linear model) *p* < 0.05.

**Table 2:** Baseline dietary intakes of participants by tertiles of energy-adjusted nut consumption at baseline examination (2006–2008): Tehran Lipid and Glucose study.

	Tertiles of Nuts Consumption			
	1	2	3	*p*
Almonds (serving/week)	0.1 ± 0.04	0.2 ± 0.04	0.8 ± 0.04	<0.001
Peanuts (serving/week)	0.1 ± 0.1	0.3 ± 0.1	0.8 ± 0.1	<0.001
Pistachios (serving/week)	0.02 ± 0.04	0.06 ± 0.04	0.31 ± 0.04	<0.001
Hazelnuts (serving/week)	0.1 ± 0.04	0.2 ± 0.04	0.6 ± 0.4	<0.001
Walnuts (servings/week)	0.7 ± 0.3	1.1 ± 0.3	7.5 ± 0.2	<0.001
Total energy (kcal/day)	2054 ± 695	2313 ± 689	2465 ± 680 ^a,b^	<0.001
Carbohydrate (% of total energy)	56.7 ± 7.8	57.8 ± 6.8	56.9 ± 6.3	0.04
Protein (% of total energy)	13.5 ± 2.5	13.6 ± 2.1	13.8 ± 2.3	0.03
Fat (% of total energy)	31.7 ± 7.8	31.0 ± 6.6	32.3 ± 6.0 ^a^	0.01
SFA (% of total energy)	10.5 ± 3.1	10.6 ± 3.0	10.9 ± 3.6	0.22
MUFA (% of total energy)	11.1 ± 3.1	10.7 ± 2.6	10.9 ± 2.4	0.11
PUFA (% of total energy)	6.2 ± 2.0	6.1 ± 2.4	6.7 ± 2.2 ^a,b^	0.01
Carbohydrate (g/day)	294 ± 5.4	335 ± 5.3	349 ± 5.4	<0.001
Protein (g/day)	70.0 ± 1.3	78.8 ± 1.3	84.2 ± 1.3	<0.001
Fat (g/day)	72.8 ± 1.5	79.5 ± 1.46	87.6 ± 1.47	<0.001
SFA (g/day)	29.4 ± 0.6	28.1 ± 0.6	29.7 ± 0.6	0.53
MUFA (g/day)	25.6 ± 0.5	26.6 ± 0.5	26.8 ± 0.5	0.45
PUFA (g/day)	15.4 ± 0.3	16.1 ± 0.4	18.2 ± 0.3	<0.001
Total fiber (g/day)	34.4 ± 21.9	37.8 ± 18.8	39.7 ± 18.3 ^a,b^	0.02
Cholesterol (g/day)	209 ± 191	227 ± 111	243 ± 121	0.78
Vegetable (g/day)	315 ± 216	359 ± 247	397 ± 242	0.11
Fruit (g/day)	273 ± 230	358 ± 270	473 ± 295 ^a,b^	0.001
Meat, poultry, fish (g/day)	44.0 ± 34.2	47.8 ± 33.7	49.4 ± 37.9	0.71
Whole grain (g/day)	73.7 ± 89.2	94.1 ± 116.3	97.0 ± 98.6	0.40
Legumes (g/day)	15.6 ± 21.8	18.2 ± 18.2	20.5 ± 20.4	0.39
Dairy products (g/day)	395 ± 268	474 ± 333	512 ± 309	0.07

SFA, saturated fatty acids; MUFA, monounsaturated fatty acids, PUFA, polyunsaturated fatty acids. Data are mean and SE, adjusted for energy intakes. *p* value determined using general linear model. ^a^ Significantly different from tertile 1 (Tukey pairwise comparison in general linear model) *p* < 0.05. ^b^ Significantly different from tertile 2 (Tukey pairwise comparison in general linear model) *p* < 0.05.

**Table 3:** Multivariate adjusted odds ratio (95% CI) for MetS across tertiles consumption of total energy-adjusted nuts and various types per se at baseline examination (2006–2008): Tehran Lipid and Glucose Study.

	Tertiles			*p* for Trend ^a^	Servings per Week
	1	2	3		
**Total nuts**					
Median intake (servings/week)	0.57	2.08	7.93		
Median (IQR) intake (g/week)	1.56 (0.66–2.80)	3.42 (2.28–5.52)	8.66 (5.33–15.76)		
Range of intake (servings/week)	≤1	2–4	≥5		
Model 1	1	0.88 (0.64–1.21)	0.55 (0.39–0.77)	0.002	0.95 (0.94–0.99)
Model 2	1	0.90 (0.68–1.29)	0.58 (0.42–0.81)	0.01	0.97 (0.94–1.05)
Model 3	1	0.94 (0.75–1.35)	0.62 (0.45–0.86)	0.01	1.02 (0.95–1.12)
Model 4	1	0.97 (0.78–1.36)	0.68 (0.44–0.91)	0.03	1.04 (0.98–0.14)
**Walnuts**					
Median intake (servings/week)	0.19	0.95	4.83		
Median (IQR) intake (g/week)	0.61 (0.05–0.83)	3.12 (1.31–7.98)	5.45 (2.69–9.16)		
Range of intake (servings/week)	≤0.5	0.5–1.4	≥1.5		
Model 1	1	0.87 (0.63–1.18)	0.61 (0.44–0.86)	0.02	0.92 (0.87–0.95)
Model 2	1	0.89 (0.69–1.20)	0.64 (0.47–0.89)	0.02	0.93 (0.90–0.95)
Model 3	1	0.91 (0.75–1.26)	0.70 (0.49–0.94)	0.03	0.94 (0.91–0.98)
Model 4	1	0.94 (0.79–1.32)	0.75 (0.53–0.98)	0.05	0.97 (0.93–0.99)
**Almonds**					
Median intake (servings/week)	0.01	0.09	0.46		
Median (IQR) intake (g/week)	0.05 (0.03–0.14)	0.29 (0.05–0.41)	0.38 (0.05–1.53)		
Range of intake (servings/week)	≤0.01	0.02–0.03	≥0.04		
Model 1	1	0.95 (0.68–1.32)	0.72 (0.53–1.33)	0.20	1.02 (0.87–1.15)
Model 2	1	1.08 (0.74–1.57)	0.79 (0.54–1.14)	0.14	1.02 (0.88–1.15)
Model 3	1	1.11 (0.76–1.61)	0.79 (0.55–1.16)	0.14	1.03 (0.89–1.20)
Model 4	1	1.13 (0.76–1.69)	0.81 (0.57–1.14)	0.25	1.04 (0.91–1.22)
**Hazelnuts**					
Median intake (servings/week)	0.01	0.05	0.23		
Median (IQR) intake (g/week)	0.05 (0.02–0.09)	0.13 (0.02–0.63)	0.13 (0.08–0.63)		
Range of intake (servings/week)	00	0.01–0.03	≥0.04		
Model 1	1	0.73 (0.53–1.03)	0.90 (0.65–1.23)	0.28	1.09 (0.92–1.29)
Model 2	1	0.89 (0.61–1.29)	1.15 (0.81–1.63)	0.27	1.06 (0.89–1.27)
Model 3	1	0.91 (0.63–1.33)	1.17 (0.81–1.69)	0.25	1.06 (0.89–1.27)
Model 4	1	0.96 (0.65–1.43)	1.23 (0.84–1.80)	0.21	1.07 (0.88–1.30)
**Peanuts**					
Median intake (servings/week)	0.00	0.06	0.46		
Median (IQR) intake (g/week)	0.07 (0.03–0.22)	0.49 (0.05–0.53)	0.49 (0.08–1.39)		
Range of intake (servings/week)	00	0.01–0.03	≥0.04		
Model 1	1	0.95 (0.67–1.34)	0.90 (0.65–1.23)	0.80	0.97 (0.86–1.10)
Model 2	1	1.04 (0.71–1.52)	1.03 (0.72–1.47)	0.90	0.98 (0.87–1.10)
Model 3	1	1.06 (0.72–1.55)	1.04 (0.72–1.50)	0.89	1.01 (0.90–1.13)
Model 4	1	1.15 (0.77–1.72)	1.04 (0.75–1.53)	0.94	1.00 (0.94–1.14)
**Pistachios**					
Median intake (servings/week)	0.00	0.03	0.14		
Median (IQR) intake (g/week)	0.09 (0.03–0.24)	0.15 (0.09–0.70)	0.73 (0.23–2.21)		
Range of intake (servings/week)	≤0.03	0.04–0.07	≥0.08		
Model 1	1	0.77 (0.55–1.08)	1.02 (0.74–1.40)	0.20	0.90 (0.50–1.64)
Model 2	1	0.82 (0.56–1.20)	1.10 (0.84–1.70)	0.25	0.97 (0.54–1.75)
Model 3	1	0.83 (0.58–1.21)	1.20 (0.89–1.74)	0.23	1.01 (0.56–1.80)
Model 4	1	0.85 (0.59–1.27)	1.24 (0.94–1.83)	0.32	1.11 (0.66–1.89)

IQR, interquartile range; ^a^ The median intake of each tertile category was assigned and then these quartile median variables were included as a continuous variable in logistic regression. Model 1 was crude. Model 2 was adjusted for family history of diabetes, age, gender, smoking, physical activity, fasting serum glucose at baseline, serum HDL cholesterol at baseline. Model 3 was additionally adjusted for total energy intake, total fiber, percent of protein, percent of carbohydrates, percent of total fat, cholesterol intake, fruit, vegetables and dairy products. Model 4 was additionally adjusted for body mass index.

**Table 4:** Stratified analyses of the consumption of energy-adjusted nuts (total) and their various types (per se) at baseline examination (2006–2008) by family history of diabetes, age and body mass index on the risk of metabolic syndrome: Tehran Lipid and Glucose Study.

	Family History of Diabetes		Age (Years)		Body Mass Index (kg/m^2^)	
	Yes	No	19–45	≥45	<25	≥25
**Total nuts**						
Tertile 1	1	1	1	1	1	1
Tertile 2	0.77 (0.33–1.78)	0.99 (0.60–1.35)	1.09 (0.73–1.62)	0.83 (0.59–1.18)	2.19 (0.91–5.28)	0.75 (0.51–1.10)
Tertile 3	0.27 (0.11–0.69)	0.86 (0.30–0.93)	0.79 (0.37–0.97)	0.49 (0.33–0.71)	1.25 (0.46–3.39)	0.43 (0.28–0.66)
*p* for trend ^a^	0.01	0.02	0.032	0.001	0.25	0.001
**Walnuts**						
Tertile 1	1	1	1	1	1	1
Tertile 2	0.73 (0.49–1.09)	1.48 (0.65–3.37)	1.11 (0.75–1.66)	0.87 (0.62–1.23)	1.14 (0.64–3.22)	0.71 (0.49–1.04)
Tertile 3	0.39 (0.25–0.61)	0.81 (0.20–0.93)	0.93 (0.46–1.17)	0.52 (0.36–0.76)	0.83 (0.27–1.93)	0.52 (0.34–0.79)
P for trend	<0.001	0.04	0.18	0.002	0.32	0.009
**Almonds**						
Tertile 1	1	1	1	1	1	1
Tertile 2	0.74 (0.31–1.74)	1.33 (0.69–1.54)	1.21 (0.79–1.85)	1.02 (0.71–1.46)	1.65 (0.65–4.21)	0.85 (0.57–1.26)
Tertile 3	0.71 (0.12–1.65)	1.31 (0.85–2.03)	0.91 (0.53–1.23)	0.79 (0.56–1.12)	1.70 (0.71–4.06)	0.59 (0.40–0.87)
*p* for trend	0.39	0.40	0.20	0.33	0.43	0.02
**Hazelnus**						
Tertile 1	1	1	1	1	1	1
Tertile 2	1.05 (0.46–2.41)	0.81 (0.53–1.23)	0.72 (0.47–1.10)	1.02 (0.71–1.46)	1.04 (0.41–2.66)	0.79 (0.53–1.17)
Tertile 3	0.88 (0.37–2.08)	1.25 (0.83–1.87)	0.95 (0.62–1.44)	1.39 (0.85–1.53)	1.07 (0.82–2.70)	1.78 (0.53–1.15)
P for trend	0.92	0.13	0.27	0.33	0.19	0.37
**Peanuts**						
Tertile 1	1	1	1	1	1	1
Tertile 2	1.21 (0.48–2.61)	1.01 (0.65–1.55)	1.22 (0.65–1.59)	0.98 (0.67–1.41)	1.42 (0.58–3.48)	0.87 (0.58–1.32)
Tertile 3	0.88 (0.36–2.13)	1.09 (0.73–1.63)	1.18 (0.58–1.35)	0.94 (0.66–1.33)	1.24 (0.42–2.53)	0.85 (0.58–1.24)
*p* for trend	0.86	0.89	0.77	0.93	0.67	0.68
**Pistachio**						
Tertile 1	1	1	1	1	1	1
Tertile 2	0.83 (0.35–1.96)	0.92 (0.59–1.41)	1.03 (0.66–1.61)	0.89 (0.59–1.21)	0.48 (0.21–1.09)	0.98 (0.52–1.06)
Tertile 3	1.03 (0.44–2.37)	1.31 (0.86–1.98)	1.30 (0.84–2.01)	0.67 (0.47–1.11)	0.51 (0.29–1.11)	1.41 (0.75–1.26)
*p* for trend	0.87	0.20	0.40	0.11	0.01	0.43

^a^ The median intake of each tertile category was assigned and then these quartile median variables were included as a continuous variable in logistic regression. Data are odds ratio (95% confidence interval). Data were adjusted for family history of diabetes, age, gender, smoking, physical activity, fasting serum glucose at baseline, serum HDL cholesterol at baseline, total energy intake, total fiber, percent of protein, percent of carbohydrates, percent of total fat and cholesterol intake, fruit, vegetables, and dairy products and BMI.

## Supplementary Materials

The following are available online at http://www.mdpi.com/2072-6643/9/10/1056/s1, Table S1: Characteristics of participants by tertiles of energy-adjusted walnut consumption at baseline (2006–2008) and after 6.2 years of follow-up (2012–2015): Tehran Lipid and Glucose Study; Table S2. Characteristics of participants by tertiles of energy-adjusted almond consumption at baseline (2006–2008) and after 6.2 years of follow-up (2012–2015): Tehran Lipid and Glucose Study; Table S3. Characteristics of participants by tertiles of energy-adjusted peanut consumption at baseline (2006–2008) and after 6.2 years of follow-up (2012–2015): Tehran Lipid and Glucose Study; Table S4. Characteristics of participants by tertiles of energy-adjusted hazelnut consumption at baseline (2006–2008) and after 6.2 years of follow-up (2012–2015): Tehran Lipid and Glucose Study; Table S5. Characteristics of participants by tertiles of energy-adjusted pistachio consumption at baseline (2006–2008) and after 6.2 years of follow-up (2012–2015): Tehran Lipid and Glucose Study.
