# Supplementary material for: Prospective Study of Nut Consumption and Incidence of Metabolic Syndrome: Tehran Lipid and Glucose Study
Source: Nutrients. 2017 Sep 23;9(10):1056. doi: 10.3390/nu9101056 (PMC5691673; doi:10.3390/nu9101056)
Supplement: Supplementary file 1 [file nutrients-09-01056-s001.pdf]

Table S1. Characteristics of participants by tertiles of energy-adjusted walnut consumption at baseline (2006‒2008) and after 6.2 years of follow-up (2012‒2015): Tehran Lipid and Glucose Study [a]

	Tertiles of Walnut Consumption			
	1	2	3	P^{b}
Participants (n)	422	422	421	
Age (y)	35.8 ± 0.6	36.7 ± 0.6	39.2 ± 0.6	<0.001
Female (%)	46.7	46.4	38.2	0.06
Physical activity (MET h-week)	40.2 ± 3.0	37.3 ± 3.0	33.9 ± 3.0	0.34
Family history of diabetes (%)	18.0	19.0	20.9	0.76
Academic degrees (%)	35.3	34.4	34.2	0.98
Occupational status, worker (%)	32.5	33.7	35.9	0.23
Current smoking (%)	12.5	11.2	8.4	0.17
BMI (kg/m^2)	23.4 ± 0.2	24.4 ± 0.2	22.4 ± 0.2	0.91
Overweight and obese (%)	56.8	60.0	60.9	0.44
Anti-hypertensive drug (%)	68.4	78.9	81.0	0.61
Anti-hyperglycemia drug (%)	83.3	80.0	85.7	0.95
Hypolipidemic drugs (%)	68.4	76.1	76.5	0.64
Systolic blood pressure (mm Hg)				
At baseline	107 ± 0.6	108 ± 0.6	108 ± 0.6	0.53
After 6.2 years	114 ± 0.7	112 ± 0.7	110 ± 0.7	0.03
Diastolic Blood pressure (mm Hg)				
At baseline	71.8 ± 0.4	71.6 ± 0.4	72.0 ± 0.4	0.82
After 6.2 years	76.8 ± 0.5	75.8 ± 0.5	75.9 ± 0.5	0.24
Fasting serum glucose (mg/dl)				
At baseline	88.9 ± 0.6	86.2 ± 0.6	84.2 ± 0.6	0.04
After 6.2 years	94.8 ± 0.9	94.5 ± 0.9	94.8 ± 0.9	0.96
Serum triglyceride (mg/dl)				
At baseline	124 ± 3.4	127 ± 3.4	124 ± 3.4	0.85
After 6.2 years	137 ± 3.5	140 ± 3.6	127 ± 2.9	0.02
Serum HDL-C (mg/dl)				
At baseline	43.3 ± 0.5	43.5 ± 0.5	44.9 ± 0.5	0.007
After 6.2 years	50.3 ± 0.6	49.3 ± 0.6	51.1 ± 0.6	0.12
Waist circumference (cm)				
At baseline	86.6 ± 0.6	87.4 ± 0.6	86.8 ± 0.6	0.73
After 6.2 years	93.9 ± 0.6	92.5 ± 0.6	91.4 ± 0.6	0.02

BMI, Body Mass Index;
Obese, BMI ≥25 kg/m^2
[a]Mean ± SE for all these values, except for variables was determined
[b] P values determined using ANOVA for continuous variables and chi-square test for categorical variables.

Table S2. Characteristics of participants by tertiles of energy-adjusted almond consumption at baseline (2006–2008) and after 6.2 years of follow-up (2012–2015): Tehran Lipid and Glucose Study [a]

	Tertiles of Almond Consumption			
	1	2	3	P^b
Participants (n)	473	316	476	
Age (y)	34.2 ± 0.5	36.1 ± 0.6	38.1 ± 0.6	<0.001
Female (%)	42.5	44.0	45.0	0.51
Physical activity (MET h-week)	35.3 ± 2.8	38.2 ± 3.5	38.3 ± 2.8	0.70
Family history of diabetes (%)	20.7	17.4	19.1	0.43
Academic degrees (%)	31.3	37.7	36.0	0.21
Occupational status, worker (%)	35.6	37.2	34.6	0.83
Current smoking (%)	9.5	12.8	10.4	0.37
BMI (kg/m^2)	23.5 ± 0.2	23.5 ± 0.2	24.9 ± 0.2	0.64
Overweight and obese (%)	59.9	58.8	58.8	0.92
Anti-hypertensive drug (%)	78.6	71.4	76.5	0.87
Anti-hyperglycemia drug (%)	77.8	77.8	79.5	0.93
Hypolipidemic drugs (%)	68.0	84.2	72.3	0.21
Systolic blood pressure (mm Hg)				
At baseline	108 ± 0.6	108 ± 0.7	107 ± 0.6	0.81
After 6.2 years	112 ± 0.7	112 ± 0.9	112 ± 0.7	0.92
Diastolic Blood pressure (mm Hg)				
At baseline	71.8 ± 0.4	71.8 ± 0.4	71.7 ± 0.4	0.98
After 6.2 years	76.2 ± 0.4	76.4 ± 0.5	76.1 ± 0.4	0.85
Fasting serum glucose (mg/dl)				
At baseline	87.1 ± 0.6	87.0 ± 0.7	85.4 ± 0.6	0.10
After 6.2 years	95.9 ± 0.8	95.6 ± 0.8	92.9 ± 0.8	0.02
Serum triglyceride (mg/dl)				
At baseline	127 ± 3.2	127 ± 3.9	121 ± 3.2	0.35
After 6.2 years	137 ± 3.3	136 ± 4.0	132 ± 3.3	0.50
Serum HDL-C (mg/dl)				
At baseline	43.6 ± 0.5	43.2 ± 0.6	44.7 ± 0.5	0.10
After 6.2 years	50.3 ± 0.6	49.6 ± 0.6	50.4 ± 0.6	0.26
Waist circumference (cm)				
At baseline	87.0 ± 0.6	87.1 ± 0.7	86.7 ± 0.6	0.86
After 6.2 years	93.4 ± 0.5	91.9 ± 0.5	90.5 ± 0.5	0.61

BMI, Body Mass Index;
Obese, BMI ≥25 kg/m^2
[a]Mean ± SE for all these values, except for variables was determined
[b] P values determined using ANOVA for continuous variables and chi-square test for categorical variables.

Table S3. Characteristics of participants by tertiles of energy-adjusted peanut consumption at baseline (2006–2008) and after 6.2 years of follow-up (2012–2015): Tehran Lipid and Glucose Study [a]

	Tertiles of Peanut Consumption			
	1	2	3	P^b
Participants (n)	422	337	506	
Age (y)	36.2 ± 0.5	36.9 ± 0.5	38.8 ± 0.6	<0.001
Female (%)	42.3	44.2	49.8	0.08
Physical activity (MET h-week)	40.5 ± 3.0	36.2 ± 3.3	35.2 ± 2.7	0.39
Family history of diabetes (%)	19.2	20.5	18.6	0.34
Academic degrees (%)	28.7	31.5	33.0	0.48
Occupational status, worker (%)	34.2	38.2	37.5	0.49
Current smoking (%)	8.6	10.8	12.4	0.21
BMI (kg/m^2)	22.4 ± 0.2	24.8 ± 0.2	22.4 ± 0.2	0.82
Overweight and obese (%)	61.4	57.3	58.7	0.48
Anti-hypertensive drug (%)	85.7	88.9	59.1	0.06
Anti-hyperglycemia drug (%)	83.3	66.7	79.5	0.41
Hypolipidemic drugs (%)	75.6	70.8	76.1	0.81
Systolic blood pressure (mm Hg)				
At baseline	109 ± 0.6	106 ± 0.7	108 ± 0.6	0.08
After 6.2 years	112 ± 0.7	111 ± 0.7	112 ± 0.7	0.48
Diastolic Blood pressure (mm Hg)				
At baseline	72.5 ± 0.4	71.0 ± 0.4	71.8 ± 0.4	0.10
After 6.2 years	76.0 ± 0.5	76.2 ± 0.5	76.3 ± 0.4	0.85
Fasting serum glucose (mg/dl)				
At baseline	86.4 ± 0.6	85.1 ± 0.6	87.3 ± 0.6	0.04
After 6.2 years	95.1 ± 0.9	93.2 ± 0.8	95.4 ± 0.9	0.19
Serum triglyceride (mg/dl)				
At baseline	123 ± 3.4	130 ± 3.8	123 ± 3.1	0.34
After 6.2 years	133 ± 3.5	141 ± 3.9	132 ± 3.2	0.13
Serum HDL-C (mg/dl)				
At baseline	43.8 ± 0.5	43.4 ± 0.5	44.3 ± 0.5	0.44
After 6.2 years	50.7 ± 0.6	49.7 ± 0.6	51.1 ± 0.6	0.51
Waist circumference (cm)				
At baseline	87.0 ± 0.6	86.4 ± 0.6	87.2 ± 0.6	0.67
After 6.2 years	92.5 ± 0.5	92.3 ± 0.5	92.1 ± 0.5	0.87

BMI, Body Mass Index;
Obese, BMI ≥25 kg/m^2
[a]Mean ± SE for all these values, except for variables was determined
[b] P values determined using ANOVA for continuous variables and chi-square test for categorical variables.

Table S4. Characteristics of participants by tertiles of energy-adjusted hazelnut consumption at baseline (2006–2008) and after 6.2 years of follow-up (2012–2015): Tehran Lipid and Glucose Study [a]

	Tertiles of Hazelnut Consumption			P^{b}
	1	2	3	
Participants (n)	454	391	420	
Age (y)	35.9 ± 0.6	37.1 ± 0.6	38.6 ± 0.5	<0.001
Female (%)	56.8	57.5	53.8	0.48
Physical activity (MET h-week)	36.2 ± 2.9	36.6 ± 3.1	38.7 ± 3.0	0.81
Family history of diabetes (%)	19.2	19.4	19.3	0.56
Academic degrees (%)	36.5	37.9	36.8	0.32
Occupational status, worker (%)	32.5	35.5	32.0	0.24
Current smoking (%)	9.7	13.9	8.6	0.04
BMI (kg/m^2)	26.7 ± 0.2	26.1 ± 0.2	26.2 ± 0.2	0.07
Overweight and obese (%)	62.1	53.9	61.1	0.31
Anti-hypertensive drug (%)	82.8	78.6	62.5	0.30
Anti-hyperglycemia drug (%)	95.3	75.0	72.7	0.20
Hypolipidemic drugs (%)	66.0	76.7	80.0	0.64
Systolic blood pressure (mm Hg)				
At baseline	108 ± 0.6	107 ± 0.7	108 ± 0.6	0.33
After 6.2 years	112 ± 0.7	112 ± 0.7	111 ± 0.7	0.37
Diastolic Blood pressure (mm Hg)				
At baseline	72.2 ± 0.4	71.4 ± 0.4	71.7 ± 0.4	0.44
After 6.2 years	76.5 ± 0.5	75.4 ± 0.5	76.5 ± 0.4	0.18
Fasting serum glucose (mg/dl)				
At baseline	86.5 ± 0.6	86.1 ± 0.6	86.7 ± 0.6	0.76
After 6.2 years	95.9 ± 0.8	94.2 ± 0.9	93.9 ± 0.9	0.22
Serum triglyceride (mg/dl)				
At baseline	127 ± 3.2	124 ± 3.5	124 ± 3.4	0.79
After 6.2 years	136 ± 3.4	135 ± 3.6	133 ± 3.5	0.76
Serum HDL-C (mg/dl)				
At baseline	43.0 ± 0.5	44.7 ± 0.5	44.2 ± 0.5	0.44
After 6.2 years	49.9 ± 0.6	51.1 ± 0.6	49.6 ± 0.6	0.20
Waist circumference (cm)				
At baseline	88.0 ± 0.6	85.7 ± 0.6	86.9 ± 0.6	0.05
After 6.2 years	93.2 ± 0.5	91.1 ± 0.5	92.4 ± 0.5	0.04

BMI, Body Mass Index;
Obese, BMI ≥25 kg/m^2
[a]Mean ± SE for all these values, except for variables was determined
[b] P values determined using ANOVA for continuous variables and chi-square test for categorical variables.

Table S5. Characteristics of participants by tertiles of energy-adjusted pistachio consumption at baseline (2006–2008) and after 6.2 years of follow-up (2012–2015): Tehran Lipid and Glucose Study [a]

	Tertiles of Pistachio Consumption			
	1	2	3	*P*[b]
Participants (n)	422	395	448	
Age (y)	38.1 ± 0.5	36.7 ± 0.6	36.9 ± 0.5	0.24
Female (%)	59.5	55.4	53.3	0.29
Physical activity (MET h-week)	38.5 ± 3.0	40.5 ± 3.1	32.8 ± 2.9	0.17
Family history of diabetes (%)	18.5	20.5	19.0	0.83
Academic degrees (%)	30.7	35.8	37.4	0.07
Occupational status, worker (%)	33.0	32.5	35.1	0.52
Current smoking (%)	11.7	12.9	7.6	0.04
BMI (kg/m^2)	27.0 ± 0.2	26.0 ± 0.2	26.4 ± 0.2	0.26
Overweight and obese (%)	41.8	42.8	38.0	0.33
Anti-hypertensive drug (%)	80.0	75.0	73.7	0.88
Anti-hyperglycemia drug (%)	96.5	88.9	76.9	0.54
Hypolipidemic drugs (%)	10.5	10.9	12.3	0.92
Systolic blood pressure (mm Hg)				
At baseline	108 ± 0.6	107 ± 0.7	108 ± 0.6	0.52
After 6.2 years	112 ± 0.7	112 ± 0.7	111 ± 0.7	0.53
Diastolic Blood pressure (mm Hg)				
At baseline	71.6 ± 0.4	71.6 ± 0.4	72.0 ± 0.4	0.80
After 6.2 years	76.2 ± 0.5	76.2 ± 0.5	76.1 ± 0.4	0.98
Fasting serum glucose (mg/dl)				
At baseline	86.7 ± 0.6	85.4 ± 0.6	87.1 ± 0.6	0.13
After 6.2 years	95.7 ± 0.9	93.3 ± 0.9	95.1 ± 0.9	0.16
Serum triglyceride (mg/dl)				
At baseline	127 ± 3.4	127 ± 3.5	121 ± 3.3	0.41
After 6.2 years	133 ± 3.5	138 ± 3.6	133 ± 3.4	0.57
Serum HDL-C (mg/dl)				
At baseline	43.4 ± 0.5	43.2 ± 0.5	45.0 ± 0.5	0.02
After 6.2 years	50.9 ± 0.6	49.6 ± 0.6	50.0 ± 0.6	0.27
Waist circumference (cm)				
At baseline	87.1 ± 0.6	86.0 ± 0.6	87.5 ± 0.6	0.27
After 6.2 years	92.6 ± 0.5	91.6 ± 0.5	92.6 ± 0.5	0.34

BMI, Body Mass Index;
Obese, BMI ≥25 kg/m^2
[a]Mean ± SE for all these values, except for variables was determined
[b] P values determined using ANOVA for continuous variables and chi-square test for categorical variables.
